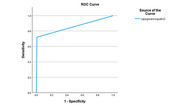# Clinical Amyloid positivity Prediction Score (CAPS) shows excellent accuracy in the COMPASS‐ND cohort

**DOI:** 10.1002/alz70861_108828

**Published:** 2025-12-23

**Authors:** Durjoy Lahiri, Shaaminy Kathir, Bruna Seixas Lima, Carlos Tyler Roncero, Howard Chertkow

**Affiliations:** ^1^ Department of Medicine, Queen's University, Kingston, ON Canada; ^2^ Queen's Health Sciences, Kingston, ON Canada; ^3^ Baycrest Academy for Research and Education, Toronto, ON Canada; ^4^ Rotman Research Institute, Toronto, ON Canada; ^5^ University of Toronto, Toronto, ON Canada; ^6^ Division of Neurology, Department of Medicine, University of Toronto, Toronto, ON Canada; ^7^ Baycrest and Rotman Research Institute, Toronto, ON Canada; ^8^ Rotman Research Institute, Baycrest Health Sciences, Toronto, ON Canada

## Abstract

**Background:**

CAPS is a simple clinical tool developed by the authors on a small Canadian cohort (*n* =48) with diagnosed clinical Alzheimer’s Disease (AD) to help clinicians predict amyloid positivity (Aβ+) (Lahiri et al, 2024). We now wished to validate CAPS on a different but similar cohort. The Comprehensive Assessment of Neurodegeneration and Dementia (COMPASS‐ND) study is a national Canadian observational study of participants clinically diagnosed with a range of neurodegenerative disorders, including Alzheimer’s syndrome and therefore presents an ideal validation opportunity.

**Methods:**

Patients with a clinical diagnosis of AD, including Subjective Cognitive Impairment (SCI) Mild Cognitive Impairment (MCI) and dementia, and known amyloid status (based on decreased csf amyloid‐beta 42 levels), from the COMPASS‐ND cohort were included. Demographic and clinical variables were compared between Aβ+ and Aβ‐subgroups, following the same framework as in Lahiri et al, 2023. CAPS was assigned as follows: cognitive decline >2 points/year on Mini Mental State Examination (MMSE)= 1 point, NPI‐Q ≥2 = 2 points, and high white matter burden (WMH)= 0 points. A total CAPS score ≥2 was considered indicative of amyloid positivity.

**Results:**

Total 86 patients fulfilled the inclusion criteria. There were no differences between the subgroups based on age, sex, and duration of illness. Aβ+ people had higher NPI‐Q scores (2 vs 0.5, *p* =0.005) and a lower baseline MMSE score (26.5 vs 28, *p* =0.009). The frequency of dementia, characterized by restriction of ADLs, was found to be higher in them (*p* <0.001). High WMH on brain MRI was reported more frequently in the Aβ‐ subgroup (50% vs 33.8%, *p* <0.001), as their cognitive decline is likely driven by non‐amyloid pathologies (mostly vascular). The frequency of people with a CAPS score of 2 or higher is significantly higher in the Aβ+ subgroup as compared to their Aβ‐ peers (72.1% vs 55.6%, *p* <0.001). CAPS ≥2 demonstrated an overall accuracy of 85.7%, sensitivity of 72.1%, and specificity of 99.9% in predicting Aβ+ status.

**Conclusion:**

This is a second and larger Canadian cohort, where CAPS was found to demonstrate excellent accuracy in distinguishing between Aβ+ and Aβ‐ subgroups. This approach allows clinicians to largely predict amyloid positivity prior to obtaining biomarkers.